# Supplementary material for: Lifespan associations of resting-state brain functional networks with ADHD symptoms
Source: iScience. 2022 Jun 26;25(7):104673. doi: 10.1016/j.isci.2022.104673 (PMC9272385; doi:10.1016/j.isci.2022.104673)
Supplement: Document S1. Figures S1–S13 and Tables S1–S3 [file mmc1.pdf]

iScience, Volume *25*

Supplemental information

Lifespan associations of resting-state brain functional networks with ADHD symptoms

Rong Wang, Yongchen Fan, Ying Wu, Yu-Feng Zang, and Changsong Zhou

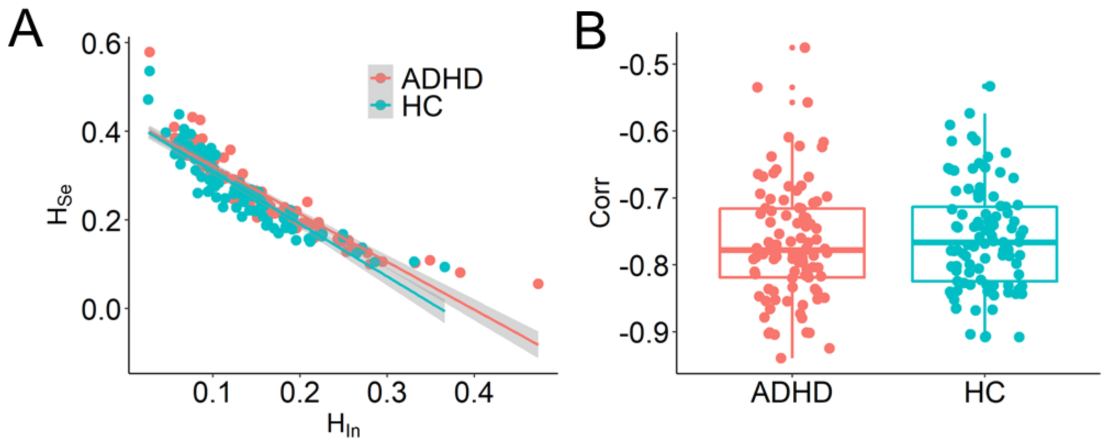

Figure S1. Highly correlated integration and segregation components, related to STAR METHODS. (A) At the whole-network level, the integration component H_{In} and segregation component H_{Se} were first calculated for each participant, and then were plotted in each group. **(B)** At the regional level, the regional integration component H_{In}^{i} and segregation component H_{Se}^{i} were first computed for each participant; and their correlation can be obtained for each participant. Then, the correlations in each group were plotted.

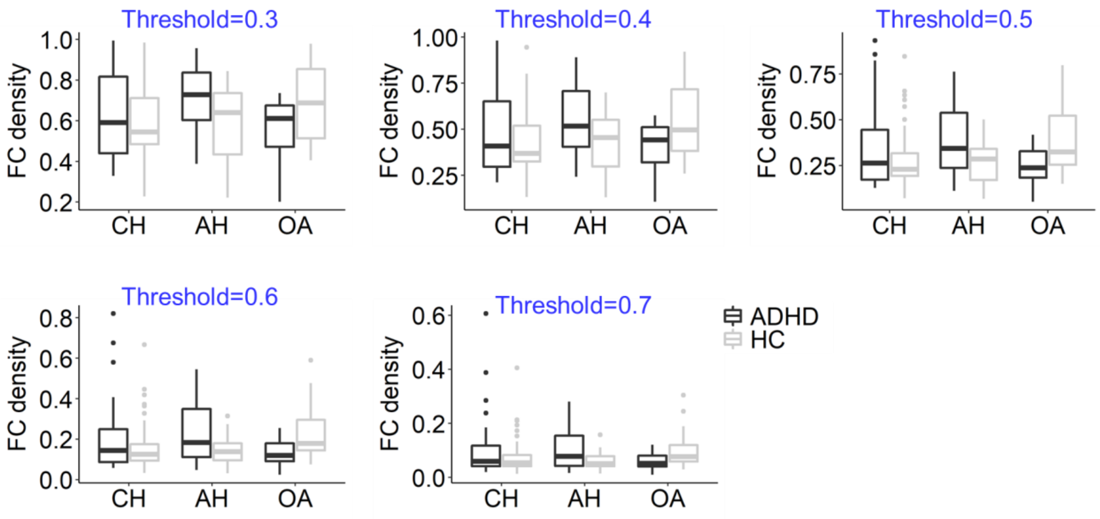

Figure S2. FC density comparisons in different thresholds, related to Figure 1. CH: 7~19 years old; AH: 21~35 years old; OA: 36~50 years old. ADHD patients have the highest density in the AH for all thresholds, indicating the nonlinear association of brain functional organization with age. However, all comparisons in FC were insignificant (p>0.05).

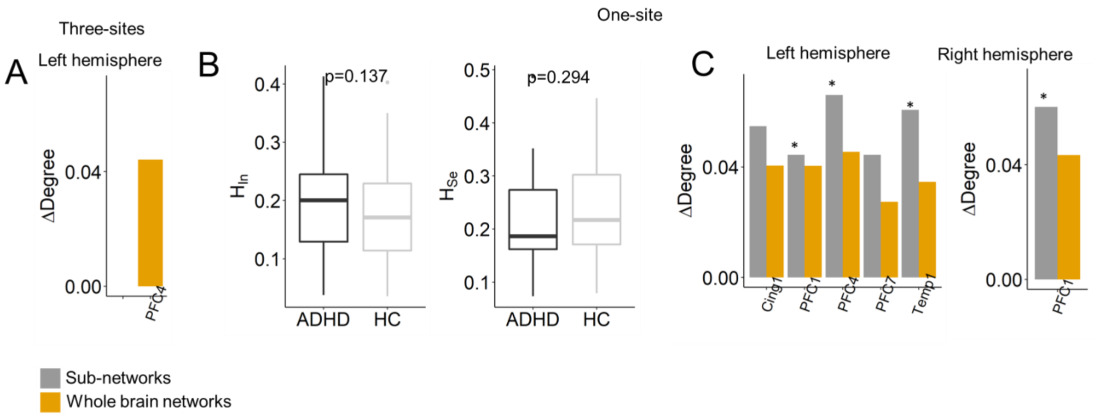

Figure S3. Alterations of regional connectivity degree in ADHD adults, related to Figure 1. (A) The degree difference between ADHD adults and HC adults was calculated in the whole-brain network for the PFC4 region. **(B)** The adult data was from the UCLA center (one-site, no harmonization of FC) and the analysis procedure was the same to that in the main text. There were insignificant difference in network segregation/integration between ADHD adults and HCs. (**C**) Alterations of regional connectivity degree in the one-site analysis. Regions with significant alterations are similar, including *PFC - prefrontal cortex*, mainly distributed in the DMN system. The PFC region has a robust alteration in the one-site and three-site analysis (multi-site correction).

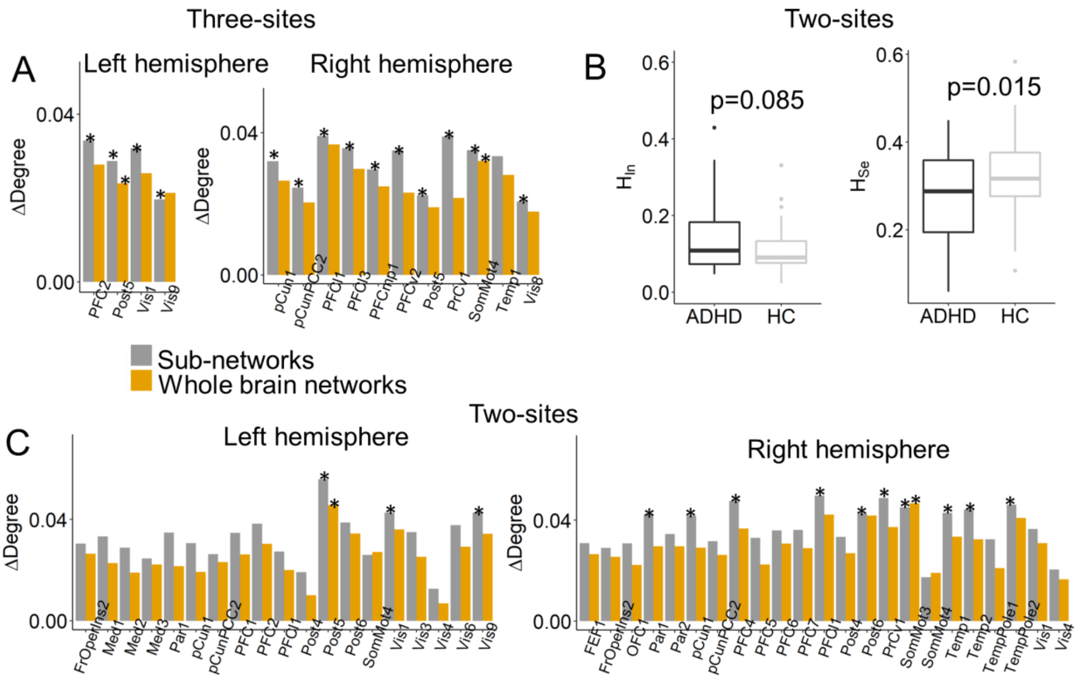

Figure S4. Alterations of regional connectivity degree in ADHD children, related to Figure 1. (A) The degree difference between ADHD children and HC children were respectively calculated in the whole-brain network and the local sub-network. * MANOVA $p<0.05$, controlling for age, sex and FD. **(B)** The children data were from the Peking and NUY centers, and the harmonization of FC was performed across the two sites. The analysis procedure was the same to that in the main text. ADHD children have significantly smaller network segregation. (**C**) Alterations of regional connectivity degree in the two-site analysis. Regions with significant alterations are similar, including *Post - posterior, Vis – visual, pCun - precuneus, PFC - prefrontal cortex, PFCl - lateral prefrontal cortex, SomMot - somatomotor, Temp – temporal*, mainly distributed in the control, dorsal attention and visual systems.

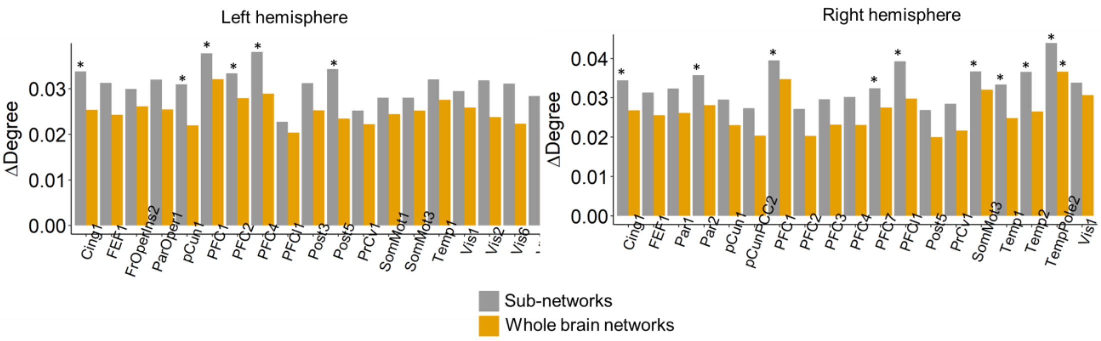

Figure S5. Alterations of regional connectivity degree in ADHD patients (three sites correction), related to Figure 1. The degree difference between ADHD patients and HCs were respectively calculated in the whole-brain network and the local sub-network. * MANOVA $p<0.05$, controlling for age, sex and FD.

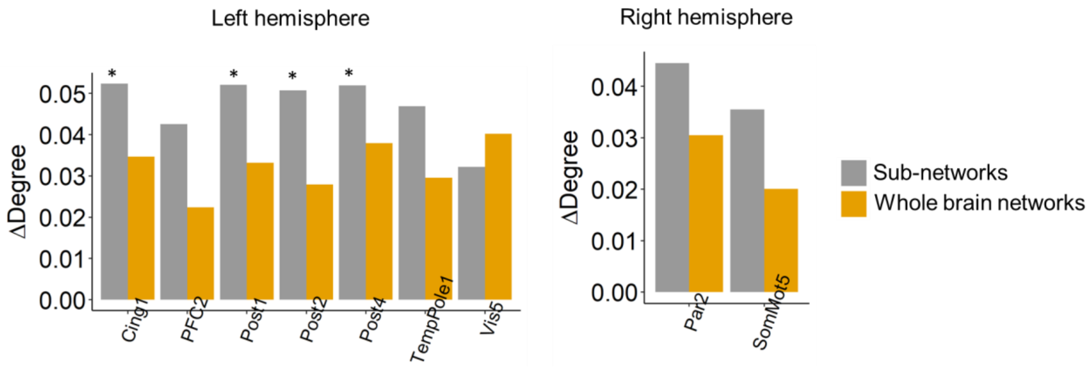

Figure S6. Comparisons of regional connectivity degree between ADHD adults and ADHD children (three-sites correction), related to Figure 1. The degree difference between ADHD adults and ADHD children were respectively calculated in the whole-brain network and the local sub-network. * MANOVA $p<0.05$, controlling for age, sex and FD. Regions with significant alterations included *Post - posterior, Cing - cingulate,* mainly distributed in the dorsal attention and control systems.

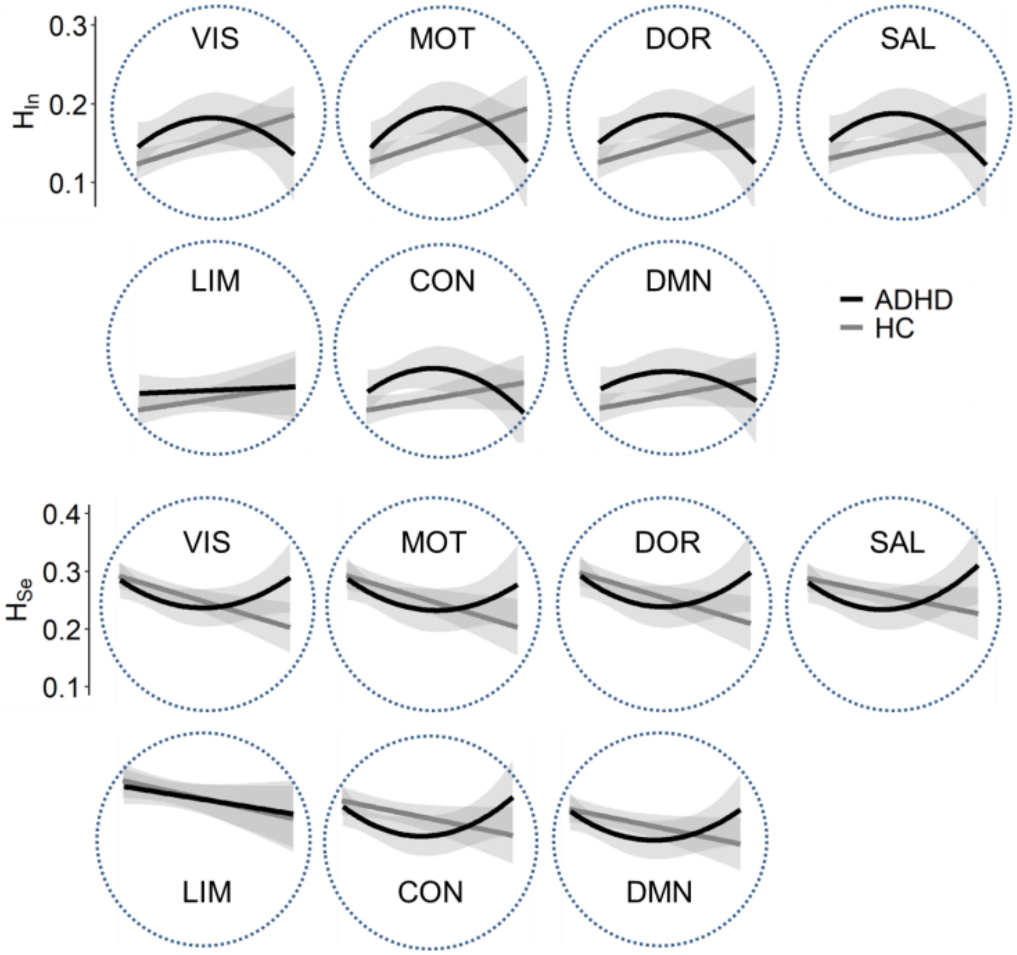

Figure S7. Lifespan associations of segregation/integration components with age in seven functional systems, related to Figure 2. The fitting lines of limbic system for segregation component between ADHD and HC have insignificant difference (p=0.588).

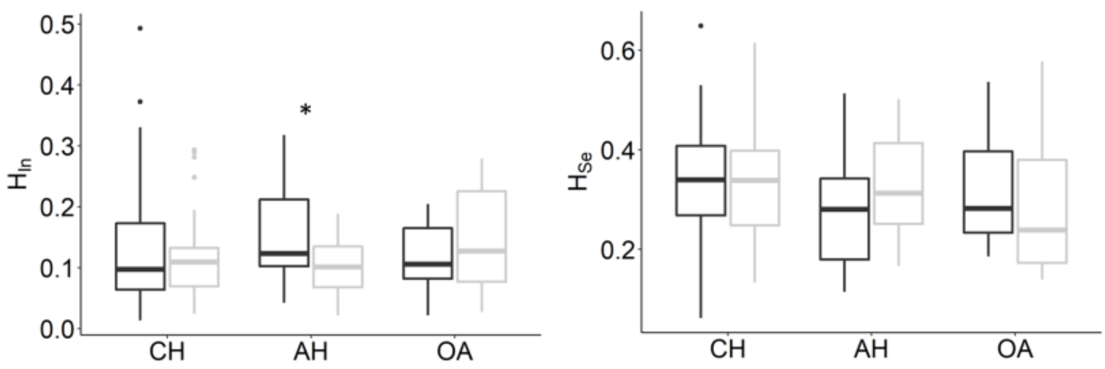

Figure S8. Segregation and integration components in the limbic system, related to Figure 2. CH: 7~19 years old; AH: 21~35 years old; OA: 36~50 years old. ADHD patients have significant higher integration component than HCs during AH (*p*=0.038, FDR corrected).

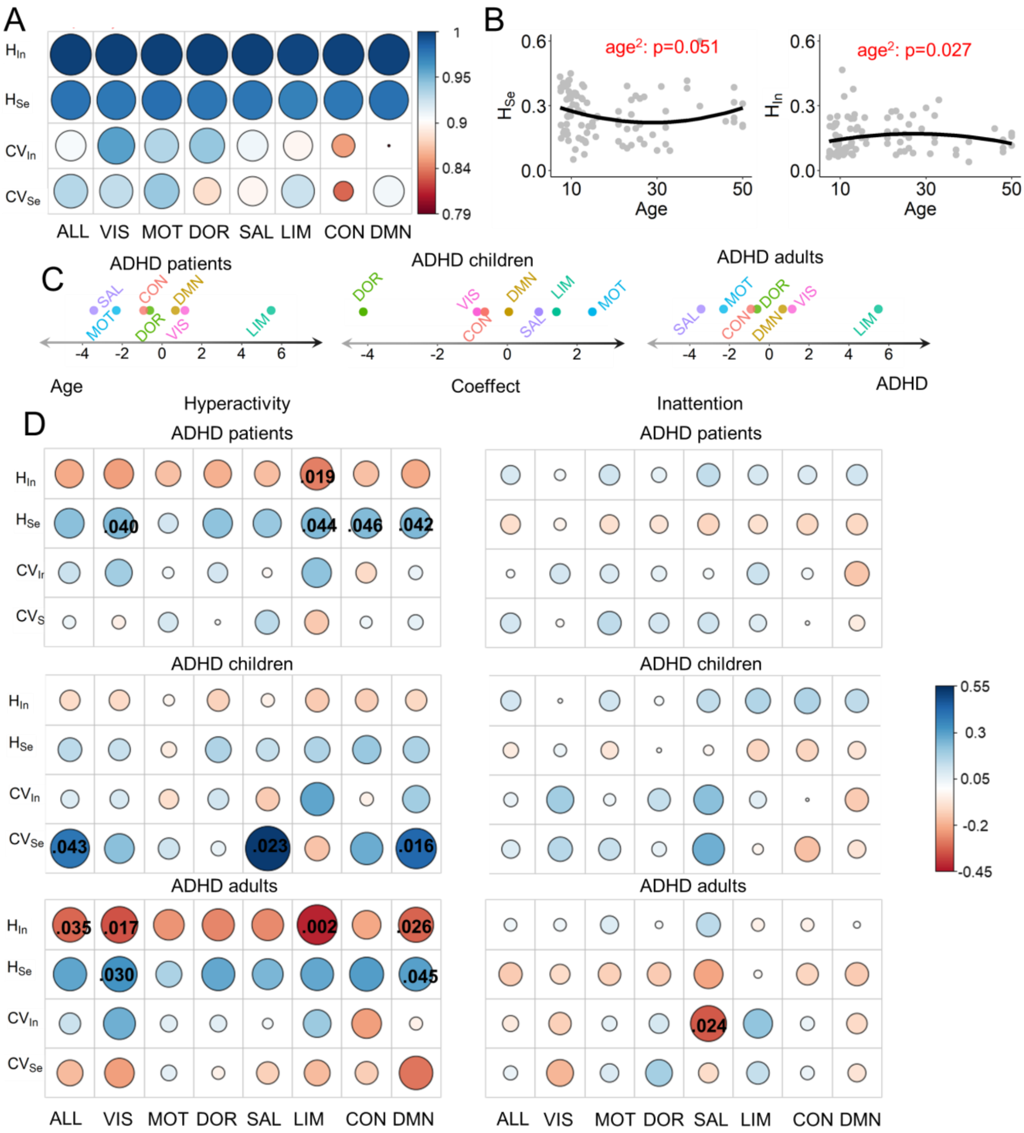

Figure S9. Main results for absolute FC, related to STAR METHODS. (A) The brain measures from absolute FC and positive FC in all participants have very high correlations. **(B)** The quadratic lifespan associations of brain network integration/segregation with age in ADHD patients. **(C)** The heterogeneous effects of age and ADHD on different functional systems. **(D)** The correlations between brain measures and hyperactivity/inattention in all ADHD patients, ADHD children and ADHD adults. Comparing this figure to Figs. 1-2 in the main text, we can see that excluding negative FC and making negative FC positive have little effect on the brain measures, lifespan association and heterogeneous effect on functional systems. However, when using absolute FC, the limbic system better predicts hyperactivity, the salient attention system better predicts inattention only in ADHD adults, and the inattention in ADHD children and all ADHD patients cannot be predicted. Thus, excluding negative FC is better for linking the brain to ADHD symptoms. A possible reason is that negative connectivity means a contrary activation between regions and indicates segregation between them. Excluding negative FC and making negative FC positive both decrease the segregation. Notably, zero connectivity can also be regarded as segregation, and positive connectivity indicates integration. Thus, making negative FC positive would generate higher integration between regions than excluding negative FC, and the relationships between brain network features and ADHD symptoms may be concealed.

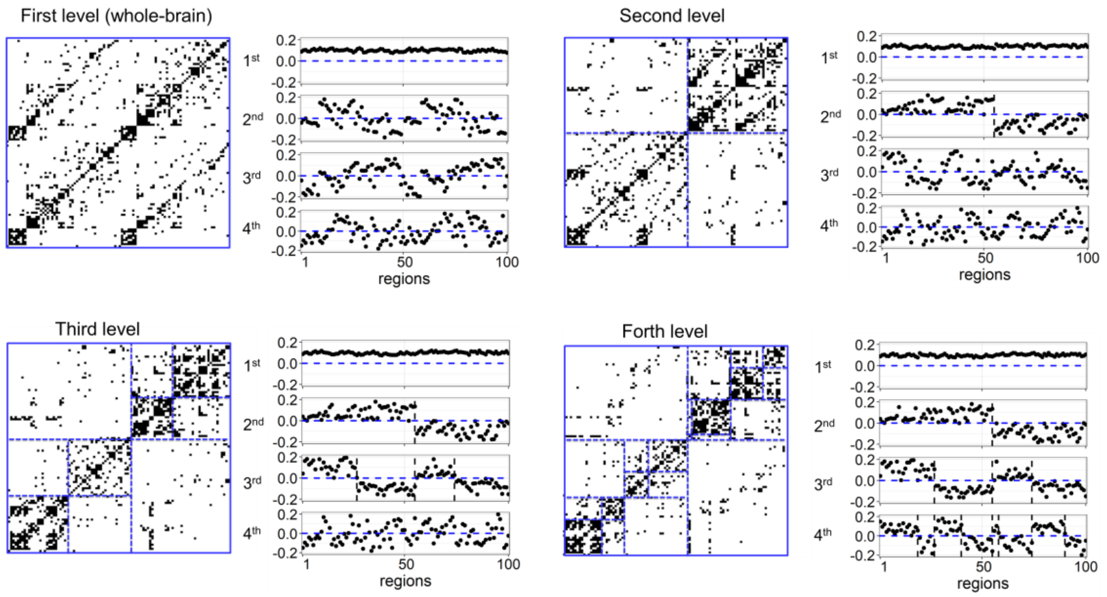

Figure S10. Hierarchical modular partition of the brain stable FC network in HCs, related to STAR METHODS. Illustration of the partition of the FC network into hierarchical modules in the first four levels. The blue dashed lines represent the boundaries of hierarchical modules suggested by the NSP method. Note that the FC network was binarized for clearer illustration, but the NSP method was performed at weighted networks. A hierarchical modular partition in the first four functional modes was also provided, where modules in each level were detected according to the positivity or negativity of eigenvector components. Note that after each partitioning step, the regions were reordered, and the order within modules remained random.

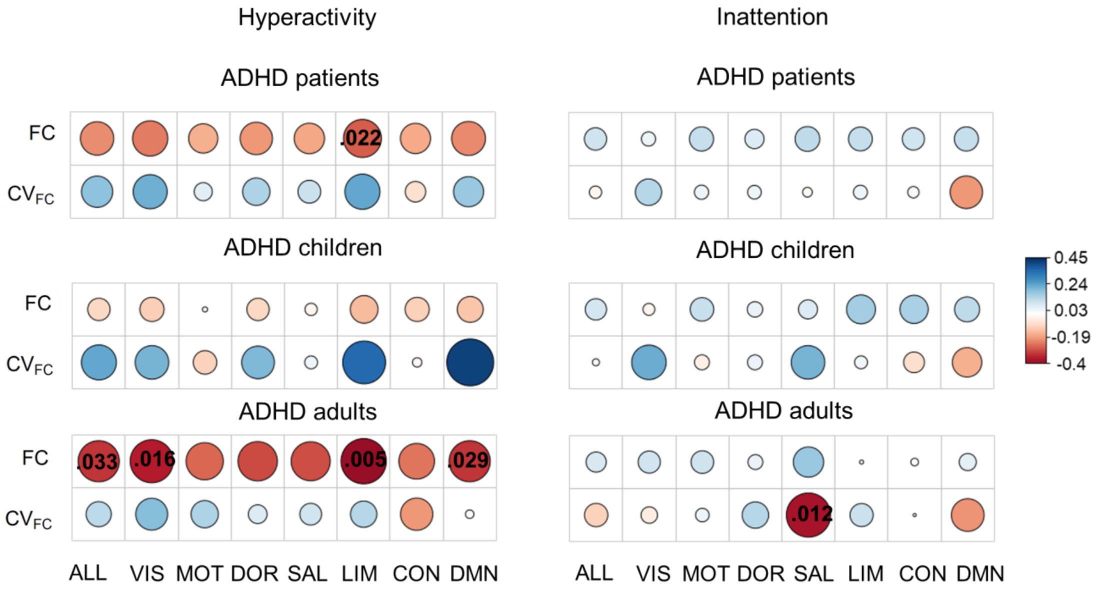

Figure S11. Multiple-regression model predicting ADHD scores based on FC in ADHD patients, related to Figures 3 and 4. The limbic system can better predict the hyperactivity in all ADHD patients and ADHD adults, but not in ADHD children. The salient attention system better predicts the inattentive severity in ADHD adults, but not in all ADHD patients and ADHD children. Thus, our NSP analysis based on eigenmodes is better in linking brain networks to ADHD scores than the classical FC analysis. Here, the CV_{FC} is the coefficient of variance based on regional degrees.

Table S1. Identifying the lifespan associations between brain functional networks and age, related to Figure 1. The form of linear regression model is $y \sim x+FD$, and it is $y \sim x^2+x+FD$ for the quadratic regression model wherein the y is the segregation or integration components and x is the age. These models were applied to the integration and segregation components of the whole-brain (ALL) network and seven systems, respectively. The likelihood ratio test (LRT) was used to identify which model is chosen. In LRT, the origin hypothesis H_0: linear model has a good fit, and the alternative hypothesis H_1: quadratic model has a better goodness of fit. If the *p*-value of LRT is smaller than 0.05, we rejected the H_0 hypothesis and chosen the quadratic model, otherwise, the linear regression model was chosen. (1) In the HC group, all *p*-values were larger than 0.05 for the whole-brain (ALL) network and seven systems, we thus chose the linear regression model to study the association of healthy brain functional organization with age. (2) In the ADHD group, both segregation and integration components in visual and limbic systems have the *p*-values larger than 0.05.

LRT *p*-value	HC		ADHD	
	H_{In}	H_{Se}	H_{In}	H_{Se}
ALL	0.850	0.716	0.055	0.041
VIS	0.545	0.591	0.084	0.054
MOT	0.483	0.889	0.025	0.125
DOR	0.780	0.441	0.046	0.046
SAL	0.765	0.674	0.037	0.036
LIM	0.339	0.741	0.397	0.276
CON	0.572	0.358	0.042	0.026
DMN	0.725	0.684	0.138	0.045

Table S2. Bootstrapping statistics on quadratic regression model in ADHD patients, related to Figure 1. The estimation coefficient for age^2 item and the corresponding 95% Confidence Interval (CI) were provided. Most of results are consistent with those from LRT, but the age^2 item has insignificant correlation with segregation/integration components in motor and limbic systems. Intersecting the results in Table S1, only limbic system is fitted with a linear regression model. The bootstrapping was performed using the "*boot*" function in **R**.

	H_{In}	H_{Se}
ALL	β=-10.27×10^{-5}; CI: (-21.14, -0.40)×10^{-5}	β=14.29×10^{-5}; CI: (1.62, 27.32)×10^{-5}
VIS	β=-9.32×10^{-5} ; CI: (-21.64, 1.33)×10^{-5}	β=12.95×10^{-5}; CI: (0.72, 25.23)×10^{-5}
MOT	β=-12.69×10^{-5}; CI: (-25.40, 0.10)×10^{-5}	β=13.47×10^{-5}; CI: (-1.30, 25.87)×10^{-5}
DOR	β=-11.75×10^{-5}; CI: (-24.20, -0.44)×10^{-5}	β=16.18×10^{-5}; CI: (1.56, 31.39)×10^{-5}
SAL	β=-11.91×10^{-5}; CI: (-23.97,-0.34)×10^{-5}	β=16.95×10^{-5}; CI: (0.89, 31.73)×10^{-5}
LIM	β=-3.99×10^{-5}; CI: (-14.75, 4.91)×10^{-5}	β=8.26×10^{-5}; CI: (-8.17, 23.72)×10^{-5}
CON	β=-10.82×10^{-5}; CI: (-21.09, -1.03)×10^{-5}	β=15.48×10^{-5}; CI: (1.67, 30.18)×10^{-5}
DMN	β=-7.70×10^{-5}; CI: (-16.85, 0.89)×10^{-5}	β=13.6×10^{-5}; CI: (1.60, 27.12)×10^{-5}

Table S3. Lifespan associations of functional connectivity with age within and between systems, related to Figure 2. The mean functional connectivity within and between systems were calculated and the LRT and bootstrapping were used to determine the lifespan association. Both methods provided similar results that functional connectivity around salient attention and control systems have the quadratic lifespan associations with age.

	VIS	MOT	DOR	SAL	LIM	CON	DMN
LRT: *p*-value							
VIS	0.320	0.080	0.120	0.115	0.344	0.029	0.113
MOT		0.229	0.050	0.015	0.205	0.003	0.065
DOR			0.161	0.029	0.201	0.031	0.061
SAL				0.034	0.191	0.026	0.131
LIM					0.444	0.229	0.913
CON						0.330	0.154
DMN							0.263
Bootstrapping: β and 95% CI							
VIS	β=-6.52×10^{-5} CI:(-19.7,6.54)×10^{-5}	β=-15.7×10^{-5} CI:(-35.6,5.5)×10^{-5}	β=-14.2×10^{-5} CI:(-32.9, -4.77)×10^{-5}	β=-12.4×10^{-5} CI:(-30.2, 4.23)×10^{-5}	β=-7.57×10^{-5} CI:(-27.7, 9.43)×10^{-5}	β=-17.3×10^{-5} CI: (-33.3, -2.33)×10^{-5}	β=-12.3×10^{-5} CI:(-28.3, 3.37)×10^{-5}
MOT		β=-8.21×10^{-5} CI:(-23.7,7.75)×10^{-5}	β=-17.4×10^{-5} CI:(-35.9, 2.03)×10^{-5}	β=-19.7×10^{-5} CI:(-36.1, -2.86)×10^{-5}	β=-11.4×10^{-5} CI:(-30.5, 7.36)×10^{-5}	β=-26.1×10^{-5} CI: (-43.9, -8.48)×10^{-5}	β=-16.7×10^{-5} CI:(-33.3, 3.34)×10^{-5}
DOR			β=-11.15×10^{-5} CI: (-26.7, 4.26)×10^{-5}	β=-18.6×10^{-5} CI:(-36.9, -1.01)×10^{-5}	β=-11.7×10^{-5} CI:(-30.3, 5.76)×10^{-5}	β=-17.2×10^{-5} CI:(-34.0, -8.27)×10^{-5}	β=-16.6×10^{-5} CI:(-32.4, 4.28)×10^{-5}
SAL				β=-16.7×10^{-5} CI:(-33.1, -6.46)×10^{-5}	β=-11.9×10^{-5} CI:(-30.0, 6.64)×10^{-5}	β=-20.4×10^{-5} CI:(-38.9, -1.26)×10^{-5}	β=-12.1×10^{-5} CI:(-27.5, 3.11)×10^{-5}
LIM					β=4.98×10^{-5} CI:(-6.89, 15.8)×10^{-5}	β=-11.0×10^{-5} CI:(-26.4, 3.87)×10^{-5}	β=-0.83×10^{-5} CI:(-14.8, 11.8)×10^{-5}
CON						β=-5.66×10^{-5} CI:(-19.1,7.82)×10^{-5}	β=-10.4×10^{-5} CI:(-25.3, 3.84)×10^{-5}
DMN							β=-7.64×10^{-5} CI:(-19.2, 3.61)×10^{-5}
